# Organic Control of Pear Psylla in Pear with Trunk Injection

**DOI:** 10.3390/insects11090650

**Published:** 2020-09-22

**Authors:** Celeste E. Wheeler, Christine Vandervoort, John C. Wise

**Affiliations:** 1 Department of Entomology, Michigan State University, East Lansing, MI 48824, USA; wisejohn@msu.edu; 2 Pesticide Analytical Laboratory, Michigan State University, 206 Center for Integrated Plant Systems, Michigan State University, East Lansing, MI 48824, USA; vanderv2@msu.edu

**Keywords:** pear psylla, trunk injection, abamectin, azadirachtin

## Abstract

**Simple Summary:**

Organic pear production is challenged, in part, by short lived effects of biopesticides when applied as foliar sprays. Trunk injection may enhance their performance by delivering the biopesticides directly to the vascular system of the tree, right where pear psylla feed. The objective of this study is to compare trunk injections to foliar sprayed applications of two insecticides, azadirachtin and abamectin, on their ability to control pear psylla in pear trees. The azadirachtin and abamectin trunk injected treatments performed equally or better than two foliar applications in the control of the pear psylla. The trunk injected trees from the first season provided a moderate level of control into the second season, one year after the injections. This study suggests that trunk injection is a superior delivery system for biopesticides used in organic pear production.

**Abstract:**

Organic production of pears is challenging in part because OMRI (Organic Materials Review Institute) approved biopesticides are short lived when applied as foliar sprays. Trunk injection is an alternative method of insecticide delivery that may enhance the performance of biopesticides for control of pear psylla. The objective of this study is to compare the efficacy of azadirachtin and abamectin in the control of pear psylla using two different application methods, airblast sprayer and trunk injection. Trunk injections of azadirachtin and abamectin were compared to airblast applications of equal labeled rates on 33-year-old Bartlett Pear trees (Pyrus communis L., var “Bartlett”). The azadirachtin and abamectin trunk injected treatments performed equally or better than the two airblast applications in the control of the pear psylla. The trunk injected trees from the first season provided a moderate level of control into the second season, one year after the injections. This study suggests that trunk injection is a superior delivery system for biopesticides used in organic pear production.

## 1. Introduction

Pear Psylla, *Cacopsylla pyricola* (Forster), is the number one insect pest to the pear industry. In fact, more than half of the money spent to control insect pests in commercial pear orchards are directed specifically at controlling pear psylla [1]. Psylla nymphs feed on sap from the trees and produce honeydew, which drips down onto the leaves and fruit. A black sooty mold grows in the honeydew, and the black color on the pears downgrades the fruit [2]. In cases where trees have been chronically heavily infested, they become stunted, reduce fruit production, and lose their leaves [3]. Pear psylla also transmit a disease that causes pear decline, which renders the tree unable to move nutrients to the roots and can cause the death of the tree [4].

Washington, Oregon, and California are the biggest US producers, with over 90% of the pears produced coming from these three states [5]. Pears grown in Michigan historically supply the food processing industry, such as Gerber/Nestle Inc. In the United States, out of over 50,000 pear bearing acres, roughly 2000 were harvested as NOP USDA (National Organic Program United States Department of Agriculture) certified organic between 2015 and 2017 [6].

Summer management of *C. pyricola* is very difficult if the overwintered population is not controlled [7]. Historically, broad-spectrum synthetic pesticides have been liberally used to control *C. pyricola*, but the pest has developed resistance [8,9]. Organic producers are limited to products that are OMRI (Organic Materials Review Institute) approved, such as oil sprays, insecticidal soaps, kaolin clay, organic pyrethrins, and azadirachtin.

Azadirachtin is a neem-based botanical insecticide that is organically approved with known activity on *C. pyricola* [10]. It is also non-toxic to birds, mammals, bees, and plants [11]. None-the-less, azadirachtin has been used only minimally in pear production because of its short residual control and higher cost. Biopesticides, in general, have shorter residual activity than synthetic insecticides because of sensitivity to environmental degradation when exposed on the surface of the plant [12]. Therefore, evaluating the potential of biopesticides for tree fruit IPM (Integrated Pest Management) should include consideration of delivery systems that will optimize their performance.

Airblast sprayers are commonly used to apply pesticides in conventional orchards. However, this method of pesticide application has drawbacks. An estimated 45% of pesticides are lost to drift and sedimentation [13,14], posing risks of non-target exposure. Reducing these risks has become a priority in Europe and the United States [15,16]. With the shift in governmental priorities towards environmental and worker protection, it is important to explore alternative pesticide application methods.

Trunk injection as an alternative pesticide application method does not pose the same non-target exposure risks as airblast application. The pesticide is delivered directly into the vascular system of the tree and is taken up through the xylem sap flow. Trunk injection is an effective application method in apple, avocado, and date trees [17,18,19,20], but no such research has been done in pear.

The objective of this study is to compare the efficacy of azadirachtin to the conventional insecticide, abamectin [21], in the control of *C. pyricola* using two different application methods, airblast sprayer and trunk injection.

## 2. Materials and Methods

### 2.1. Field Plots and Treatment Compounds

This experiment targeted natural populations of *C. pyricola* at the MSU Trevor Nichols Research Center in Fennville, MI, USA (latitude 42.5951°: longitude −86.1561°). The two insecticides used were azadiraachtin (Azasol^TM^, Arborjet Inc., Woburn, MA, USA), and abamectin (Agri-Mek^TM^, Syngenta AG, Greensboro, NC, USA). Treatments were made on 33-year-old Bartlett Pear trees (Pyrus communis L., var “Bartlett”) with single tree replicates and 4 replicate trees per treatment in a randomized complete block design. 

Rates of compounds were based on labeled rates for use in pears. Trunk injections were applied with the equivalent amount of active ingredient per tree based on orchard tree spacing (Table 1).

### 2.2. Trunk Injection

Treatment injection applications were made 7 days after petal fall (23 May 2017). Injections were performed using an Arborjet Tree IV with 4 portals equally spaced along the circumference of each trunk. The injection equipment included the Arborjet Tree IV^TM^ kit (Tree IV, #4 arbor plugs, and plug tapper) (Arborjet Cleanjet^TM^, Arborjet, Inc., Woodburn, MA, USA), hammer, cordless drill, and a 0.95 cm wood drill bit. First, 4 holes were drilled into the pear trunk approximately 5 cm deep, 90° horizontal from the trunk, and 30 cm above the ground, spaced as equally as possible while strategically placing under the main scaffold branches of the tree to distribute maximum compound volume throughout the canopy. Next, the plugs were tapped into place deep enough thus that the outside rim of the plug was just below the bark. At this stage, the tree was ready for injection. 

Before each injection, the Tree IV was cleaned with a sanitizing solution (Arborjet Cleanjet^TM^, Arborjet, Inc., Woodburn, MA, USA) and water to rinse out residues. The insecticide was measured out and diluted into distilled water, thus that the final volume was 500 mL. The compound was then poured into the Arborjet injector holding tank. The needles were inserted into the plugs, and the compound was injected via a hand-operated pressurized pump (Arborjet, Inc., Woodburn, MA, USA, part number 070-2025) into the tree. 

### 2.3. Foliar Application

Foliar applications were performed using an FMC 1029 airblast sprayer (Jonesboro, AK, USA) in 935 L/ha of water (100 GPA, gallons per acre). The 1st applications were made on the 23 May 2017 and 30 May 2018. A 2nd application of the foliar treatments was made on 23 June 2017 and 17 July 2018 after psylla nymphs reached an action threshold of 1 nymph per 3 leaves [1]. Individual trees were sprayed by using the nozzles on one side of the airblast sprayer only to apply the treatment on one side of the tree at a time. Foliage on both sides of the tree were thoroughly covered by the output of the sprayer.

### 2.4. Field Evaluations

To evaluate the *C. pyricola* numbers, field evaluations were conducted every 2 weeks in the 2017 and 2018 seasons. Samples of 50 leaves were taken (randomly throughout the entire tree—high, low, shielded, exposed, and all 4 quadrant areas) for each replication. Pear psylla eggs and nymphs were counted using a stereomicroscope. In 2017, *C. pyricola* nymph and egg evaluations were made on the 31 May, 12 June, 19 June, 28 June, 14 July, and 21 July. In 2018, *C. pyricola* nymph and egg evaluations were made on the 18 June, 16 July, 25 July, and 8 August. All the field evaluations on the 2017 injection trees continued into the 2018 season with field evaluation dates occurring June through mid-August.

At the end of the season, on 17 August 2017, a 2 min count for sooty mold occurred. Each tree was surveyed for 2 min and leaves with sooty mold were counted. This evaluation was used to quantify the negative impact of psylla honeydew on leaves at the end of the season.

### 2.5. Residue Sample Collection and Preparation

Pear leaf and fruit samples were taken for residue analysis. Leaf samples were collected on 1, 7, 14, 28, 56, and 84 days after treatment (DAT). Fruit samples were taken on 7 and 84 days after treatment. For the leaf samples, 40 leaves were randomly sampled from each replication (approximately 25 g). For the fruit samples, 5 pears were randomly sampled from each replicate tree. Each pear was diced into half-inch squares and homogenized in a bowl, and 25 g were taken from this sample. Pears taken at 7 DAT were not as large as the 84 DAT fruit, therefore, after dicing and homogenizing the 7 DAT samples, most of the homogenized sample was used to make 25 g. Samples were weighed and held in 50 mL of dichloromethane (DCM) until processing. Samples were mixed with 4 g magnesium sulfate and 1 g sodium chloride and allowed to sit for 48 h in a 4 °C walk-in cooler. The DCM was then filtered through a funnel lined with filter paper and 10 g of sodium sulfate to remove water and allowed to evaporate under a hood for 4–12 h. We added 2 mL acetonitrile to the evaporated jars and swirled for 90 s to ensure maximum uptake of the dried pesticide residue. The acetonitrile solution was then analyzed on a HPLC (High Performance Liquid Chromatography) utilizing a previously reported method [22,23]. 

### 2.6. Residue Sample Analysis

The residue levels were quantified using a waters 2695 separator module HPLC equipped with a Waters MicroMass ZQ mass spectrometer detector (Waters, Milford, MA, USA), and a C18 reversed-phase column 50 by 3.0 mm bore, 3.5 µm particle size (Waters, Milford, MA, USA).

The mobile phase, solvent A, was water with 0.1% formic acid, and solvent B was acetonitrile with 0.1% formic acid and was initially held at 80% solvent A and 20% solvent B and followed by a gradient. The column temperature was 40 °C.

Monitored ions for abamectin were 158.3 and 886.7 m/z (Da). The HPLC level of quantification was 0.0023 mg/kg^−1^ of a.i., and level of detection was 0.001 mg/kg^−1^. By using the above described extraction method, the mean parent compound recovery from 4 pear leaf samples (each 100 g) treated only with standard abamectin solution (0.046 mg/kg^−1^), then agitated and left to dry, was 73%. The results have not been corrected for abamectin recovery.

Monitored ions for azadirachtin were 685.4 and 703.4 m/z (Da). The HPLC level of quantification was 0.015 mg/kg^−1^ of a.i., and level of detection was 0.005 mg/kg^−1^. By using the above described extraction method, the mean parent compound recovery from four pear leaf samples (each 100 g) treated only with standard azadirachtin solution (0.030 mg/kg^−1^), then agitated and left to dry, was 83%. The results have not been corrected for azadirachtin recovery.

### 2.7. Statistical Analysis

The psylla field evaluation data were analyzed using a repeated-measures analysis as a 2-way RCBD using the PROC MIXED procedure in SAS 9.4 (SAS Institute, Cary, NC, USA, 2013). The following statistical model was fitted to the data:

PearPsylla_ijk_ = µ + Block_j_ + Trt_i_ + ε_1ij_ + Time_k_ + (Trt_i_ × Time_k_) + ε_2ijk_, (1)

I = 1,2,3,4,5   j = 1,2,3,4   k = 1,2,3,4,5,6

The degrees of freedom for the model components were equal to 1 for the grand mean (µ); 4 − 1 = 3 for Block_j_; 5 − 1 = 4 for Trt_i_; 3 × 4 = 12 for the residual ε_1ij_; 6 − 1 = 5 for Time_k_; 4 × 5 = 20 for (Trt_i_ × Time_k_); and 120 − (1 + 3 + 4 + 12 + 5 + 20) = 75 for the residual ε_2ijk_.

The normality assumption was assessed by checking normal probability plots and histograms of residuals. The equal variance assumption was assessed by checking plots of residual v. predicted values, side by side box plots, and Levene’s test.

The data indicate that as time goes on, the variability in the variances increases, making it necessary to fit a variance-covariance structure with unequal variances. When treatments were significant, all pairwise comparisons among the treatment means were analyzed. PROC GLIMMIX SAS 9.4 (SAS Institute, Cary, NC, USA, 2013) was run to generate a plot of pear psylla least-squares means for treatment by evaluation day sliced by treatment and adjusted for Tukey-Kramer honestly significant difference (*p* ≤ 0.05).

## 3. Results

### 3.1. Field Evaluations

#### 3.1.1. 2017 Season

**Figure 1:**
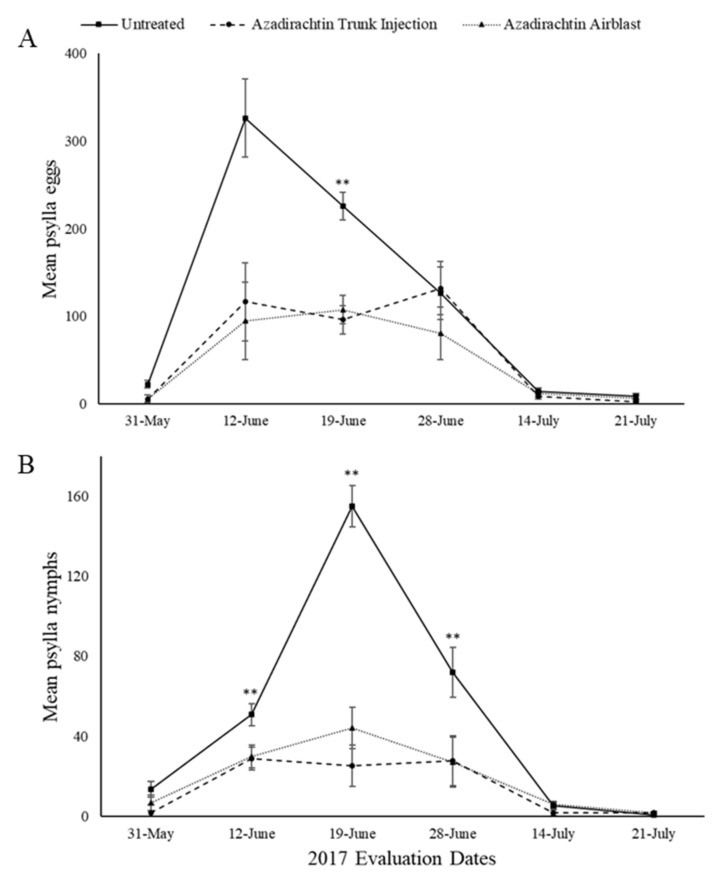
Mean number of pear psylla eggs (**A**) and nymphs (**B**) per 50 leaves in 2017 evaluations for the untreated and azadirachtin treatments. Values with ** above them represent a significant difference (*p* ≤ 0.05) between the untreated and both injection and airblast treatments.

**Figure 2:**
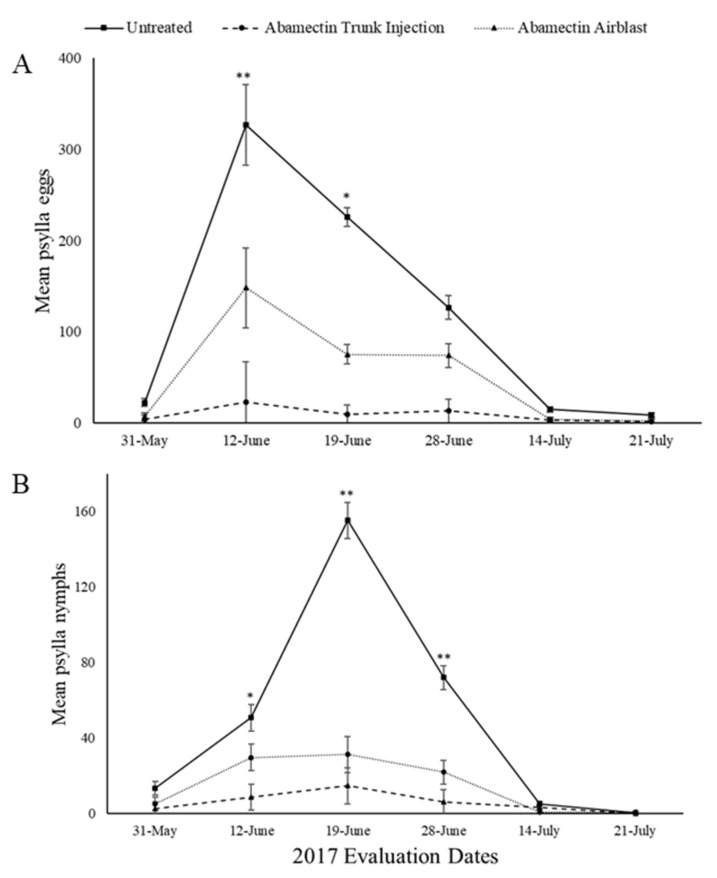
Mean number of pear psylla eggs (**A**) and nymphs (**B**) per 50 leaves in 2017 evaluations for the untreated and abamectin treatments. Values with * above them represent a significant difference (*p* ≤ 0.05) between the untreated and the trunk injected treatment only. Values with ** above them represent a significant difference (*p* ≤ 0.05) between the untreated and both injection and airblast treatments.

The overall treatment effect for mean *C. pyricola* eggs was significant (F = 16.01, Num df = 4, Den df = 23.4, *p* < 0.0001). Differences of treatment by evaluation day LS (Least Squares) means sliced by treatment indicated a total of 5 significant differences between treatments (Figure 1A and Figure 2A). 

The overall treatment effect for mean *C. pyricola* nymphs was significant (F = 39.46, Num df = 4, Den df = 22, *p* < 0.0001). Differences of treatment by evaluation day LS means sliced by treatment indicated a total of 11 significant differences between treatments (Figure 1B and Figure 2B). 

Azadirachtin injected and azadirachtin airblast treatments significantly reduced the number of eggs as compared to the untreated on 19 June 2017 (azadirachtin injection *p* = 0.003, azadirachtin airblast *p* = 0.0012) (Figure 1A). 

Azadirachtin injected treatments significantly reduced the number of nymphs as compared to the untreated on 12 June 2017 (*p* = 0.0313), 19 June 2017 (*p* < 0.0001), and 28 June 2017 (*p* = 0.0074). Azadirachtin airblast treatments significantly reduced the number of nymphs as compared to the untreated on 12 June 2017 (*p* = 0.0385), 19 June 2017 (*p* < 0.0001), and 28 June 2017 (*p* = 0.0066) (Figure 1B).

Abamectin injected treatment significantly reduced the number of eggs as compared to the untreated treatment on 12 June 2017 (*p* = 0.0119) and 19 June 2017 (*p* = 0.0003). Abamectin airblast treatment significantly reduced the number of psylla eggs as compared to the untreated check on 12 June 2017 (*p* = 0.0022) (Figure 2A).

Abamectin injected treatment significantly reduced the number of nymphs as compared to the untreated treatment on 12 June 2017 (*p* = 0.0112) and 19 June 2017 (*p* < 0.0001), and 28 June 2017 (*p* = 0.0098). Abamectin airblast treatment significantly reduced the number of *C. pyricola* nymphs as compared to the untreated check on 19 June 2017 (*p* < 0.0001) and 28 June 2017 (*p* = 0.0032) (Figure 2B).

The sooty mold evaluation showed significantly fewer leaves infected on treated trees compared to untreated trees (F = 17.92, NumDF = 4, DenDF = 12, *p* < 0.0001). All treatments averaged less than 16 leaves with sooty mold, significantly fewer than those in the untreated check (Table 2).

#### 3.1.2. 2018 Season

2017 injected trees were evaluated in 2018 along with the 2018 airblast applicated treatments.

**Figure 3:**
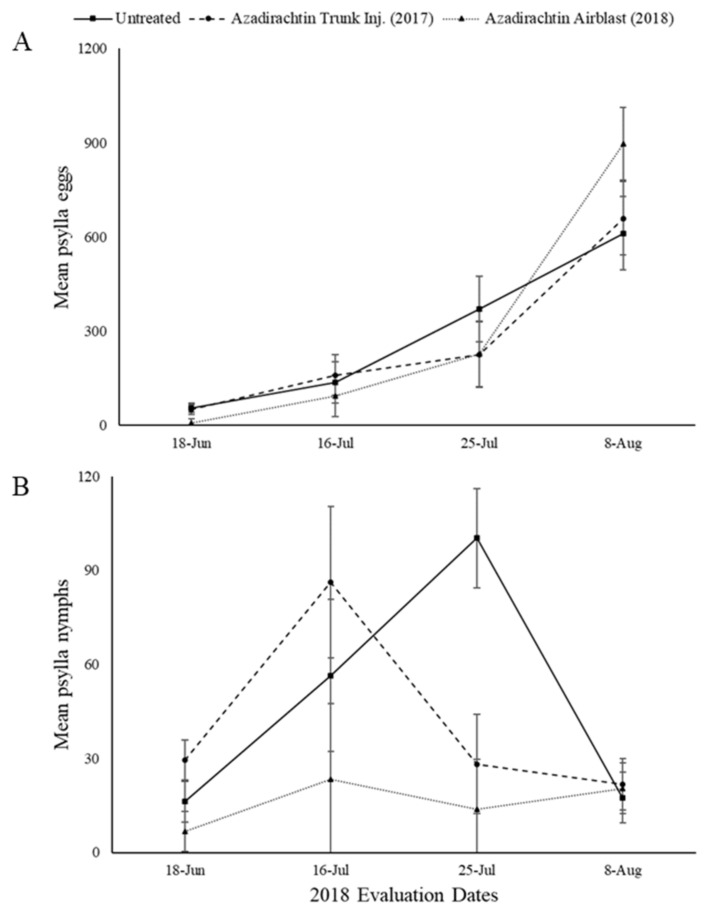
Mean number of pear psylla eggs (**A**) and nymphs (**B**) per 50 leaves in 2018 evaluations for the untreated and azadirachtin treatments. There was no significant difference found between any of the treatments (*p* ≤ 0.05).

The overall treatment effect for mean *C. pyricola* eggs was not significant for azadirachtin (F = 0.11, Num df = 2, Den df = 9.01, *p* = 0.8972) (Figure 3A).

The overall treatment effect for mean *C. pyricola* nymphs was not significant for azadirachtin (F = 3.12, Num df = 2, Den df = 9.71, *p* = 0.0897) (Figure 3B).

**Figure 4:**
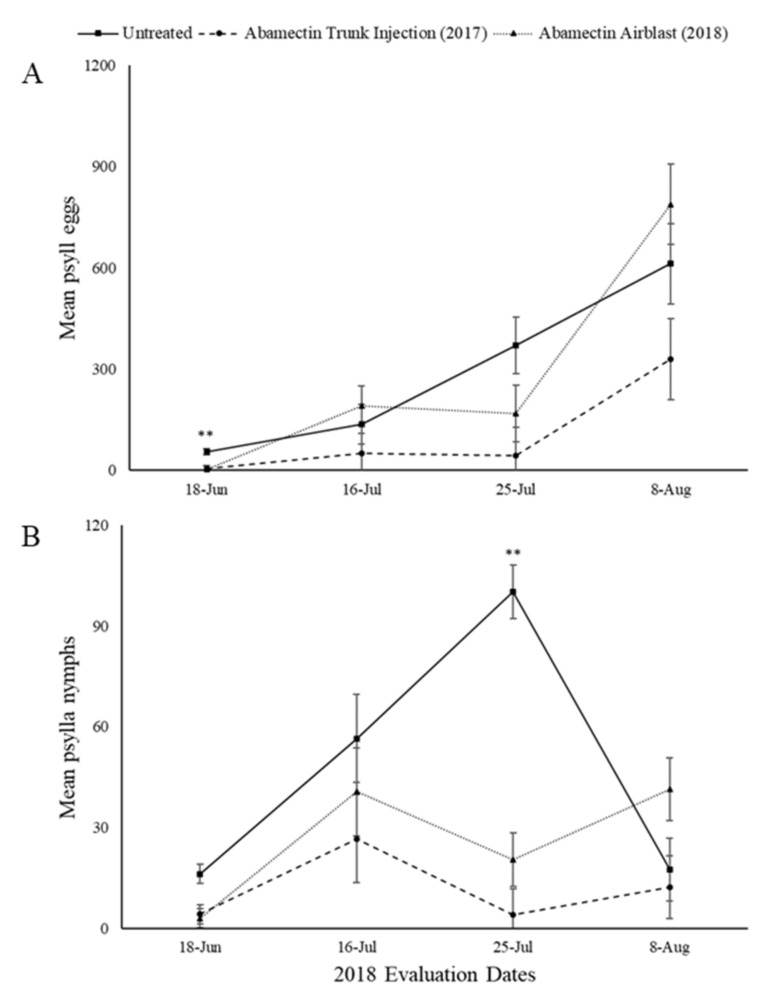
Mean number of pear psylla eggs (**A**) and nymphs (**B**) per 50 leaves in 2018 evaluations for the untreated and abamectin treatments. Values with ** above them represent a significant difference (*p* ≤ 0.05) between the untreated and both injection and airblast treatments.

The overall treatment effect for mean C. pyricola eggs was significant for abamectin (F = 6.57, Num df = 2, Den df = 12.7, *p* = 0.0109) (Figure 4A). Differences of treatment by evaluation day LS means sliced by treatment indicated a total of 2 significant differences between treatments.

Abamectin treatments significantly reduced *C. pyricola* nymphs (F = 13.8, Num df = 2, Den df = 11.2, *p* = 0.002) (Figure 4B). Differences of treatment by evaluation day LS means sliced by treatment indicated a total of 2 significant differences between treatments.

Abamectin trunk injection and airblast treatments reduced the number of eggs on 18 June 2018 (*p* < 0.05) relative to the untreated control but there was no effect of treatment on the number of eggs the rest of the season (Figure 4A). Without treatment, the number of nymphs peaked in mid-summer, but populations were reduced significantly (*p* < 0.0001) by both insecticide treatments on 25 July 2018 (Figure 4B). 

### 3.2. Residue Profiling

Azadirachtin-treated fruit samples had only one detection, which was in the trunk injection treatment for samples taken 7 days after application (Table 3). No residues were detected in abamectin treated fruit samples (Table 4). No residues were detected above the MRL(Maximum Residue Level) for fruit (abamectin MRL = 0.02 ppm, azadirachtin was exempt from the tolerance requirement) [24].

**Figure 5:**
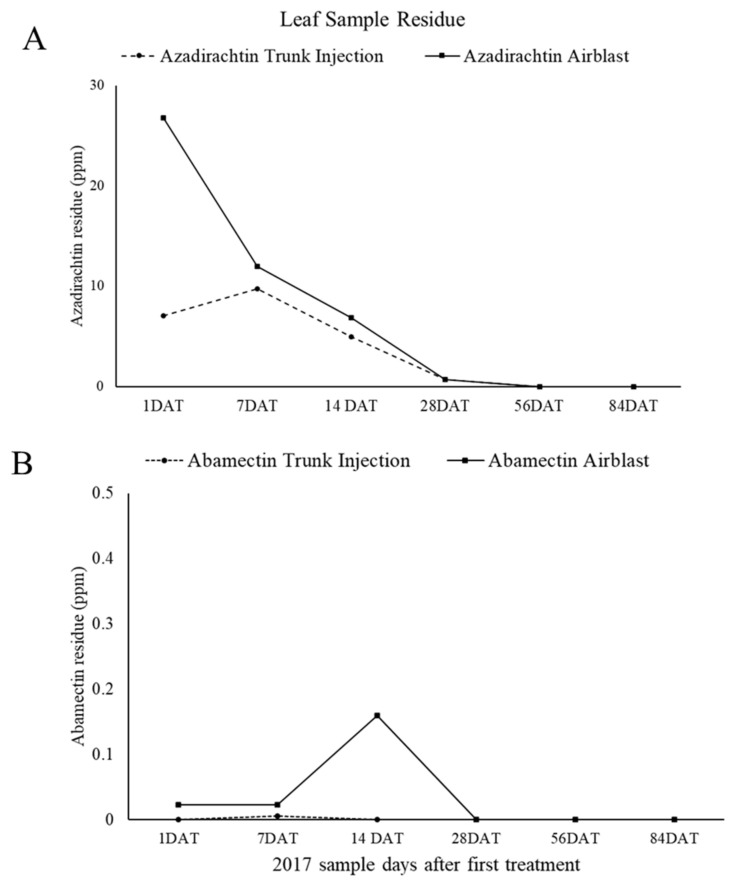
Mean residue from leaf samples taken in 2017 azadirachtin trunk injection and airblast treatments (**A**) and of abamectin trunk injection and airblast treatments (**B**). Samples were taken 1, 7, 14, 28, 56, and 84 days after treatment (DAT). Residue is presented as ppm (µg/mL) above the level of detection (LOD) (abamectin LOD = 0.001 ppm) (azadirachtin LOD = 0.005 ppm). No residue was detected after 28 DAT for both abamectin and azadirachtin.

Leaf sample residues for azadirachtin were highest following applications and steadily decreased throughout the season (Figure 5A). All azadirachtin residues were below 30 ppm, and no residue was detected beyond 28 days after treatment. Leaf sample residues for abamectin were relatively low, with all residue detections below 0.25 ppm, and most detections below 0.1ppm (Figure 5B). 

## 4. Discussion

This study contributes new information on how trunk injection of biopesticides may enhance the control of *C. pyricola* in pear production. A single injection of azadirachtin provided season-long control, and two seasons control following a single injection of abamectin. Most importantly, one trunk injected application of either product resulted in the same or better control than four foliar sprays over two seasons. 

Azadirachtin is an antifeedant, repellant, and insect growth regulator (IGR) [25,26,27]. Azadirachtin affects the morphogenesis, ovarian development, fecundity, egg viability, and molting of psylla through the endocrine system [25]. The injection data reflected the IGR effect with significantly lower nymphs starting just a week after injection. The antifeedant and repellant properties of azadirachtin likely played a role in lowering psylla numbers as well.

Leaf residue levels dropped rapidly after the first azadirachtin foliar spray, whereas azadirachtin showed a more uniform pattern of persistence in the trunk injected trees. This makes sense as the tree can store azadirachtin within leaf tissue as a metabolite without changing its biological effect [28,29]. Azadirachtin on the surface of the plant, however, breaks down quickly under ultraviolet light [30,31]. The biological activity is significantly reduced around 200 h of exposure to UV (Ultraviolet) light [30]. The antifeedant potency is rapidly decreased with exposure to sunlight [32]. The half-life of azadirachtin was found to be less than an hour [31]. Sun exposure degradation may explain why the residues for the airblast application declined rapidly, and why airblast applied azadirachtin was not effective for as long as trunk injected azadirachtin. 

Abamectin is a neurotoxin, permanently opening the glutamate-gated chloride channels and inhibiting the nerve and muscle cell communication. When ingested, the psylla is affected by uncoordinated movement, paralysis, starvation, and ultimately death [33]. The neurotoxicity effects the nymph stage most, stopping life before reproduction occurs, and our data reflect this. Egg numbers stayed quite low, never ramping up to a peak in egg production, and always staying flat line at a low level. Nymph numbers were consistently below the action threshold (0.3 nymphs/leaf) for the abamectin trunk injected treatments. While airblast treatments were not statistically different from the trunk injected treatments, they were consistently above the nymph action threshold, meaning that farmers would take action at that point to protect their crops by making another insecticide application. If a second application can be avoided, time and money can be saved, and less pesticide introduced into the environment. 

Abamectin rapidly degrades when exposed to light [33,34]. Abamectin products used in tree fruit production are formulated for foliar application, for which horticultural oils are recommended to move the active ingredient into the leaf tissue. Foliar application of abamectin with horticulture oil is generally expected to control pear psylla for half the season. Trunk injected abamectin provides season long control and may be further protected from degradation within the tree canopy. This likely explains why the injected treatment was superior in its ability to control pear psylla. 

This is the first modern study in the United States demonstrating the potential for using trunk injection of biopesticides in pear trees. Our study showed that injected insecticides achieved a high level of control over *C. pyricola* for two seasons. Likewise, this has been documented in apple trees for similar phloem-feeding insects such as potato leafhopper and rosy apple aphid [19,35,36,37]. Residues in pear fruit were extremely low and often zero, well below the MRL allowed by the US EPA, similar to other studies in apple [37].

## 5. Conclusions

In conclusion, our study shows that the trunk injection of insecticides to control *C. pyricola* in pear trees has many promising aspects for future pear production. For organic production in particular, which is reliant on biopesticides, trunk injection can enhance the control of *C. pyricola*. First, one injection provides a high level of control of *C. pyricola* for two seasons using 75% less insecticide than what is needed with airblast application. Second, the insecticide is delivered directly to the feeding psylla through the sap of the tree, therefore, it is not lost to drift, runoff, or subjected to photodegradation. This reduces production costs for farmers and saves the environment from unnecessary non-target exposure. Thirdly, detections of pesticide residues for abamectin and azadirachtin were zero for fruit at harvest. This reduces exposure and dietary risks to farmworkers and consumers. 

Trunk injection works well in reducing *C. pyricola* in pear. Future efforts to make the injection process more efficient will lead to economic feasibility for farmers to adopt. Currently, the trunk injection systems require 2 to 5 min per tree to deliver crop protection materials. Economic viability may depend on reducing the time and labor needed to cover acres of orchard crops.

## Figures and Tables

**Table 1:** Treatment rates for pear trunk injection and airblast application comparison at the Trevor Nichols Research Center, Fennville, MI in 2017 and 2018.

Treatment/Application Method	Trade Name	Active Ingredient	Application Rate	Active Ingredient per Tree
Untreated Control	-	-	-	-
Abamectin/Trunk Injection	Agri-Mek 0.15 EC	abamectin	2.40 mL/tree	0.04 g
Abamectin/Airblast	Agri-Mek 0.7 SC	abamectin	0.29 L/ha	0.04 g
Azadirachtin/Trunk Injection	Azasol 6%	azadirachtin	4.0 g/tree	0.24 g
Azadirachtin/Airblast	Azasol 6%	azadirachtin	2.45 kg/ha	0.24 g

**Table 2:** Mean leaves with black sooty mold per treatment after conducting a 2 min visual count on 17 August 2017.

Treatment/Application Method	Mean Leaves with Black Sooty Mold
Untreated	71.5 a
Abamectin/Trunk Injection	15.8 b
Abamectin/Airblast	10.3 b
Azadirachtin/Trunk Injection	8.5 b
Azadirachtin/Airblast	14 b

a, b: Means followed by the same letter do not significantly differ (*p* < 0.0001, *p* ≤ 0.05, Tukey-Kramer honestly significant difference).

**Table 3:** Mean azadirachtin residue on fruit taken 7 days and 84 days after the first treatment (DAT). Residue (ppm) reported above the level of detection (0.005 ppm), or below the level of detection (nd).

Treatment/Application Method	7 DAT (Days after First Treatment)	84 DAT
Untreated	nd	nd
Azadirachtin/Trunk Injection	1.507	nd
Azadirachtin/Airblast	nd	nd

**Table 4:** Mean abamectin residue on fruit taken 7 days and 84 days after the first treatment (DAT). No residue was detected (nd) above the level of detection (0.001 ppm).

Treatment/Application Method	7 DAT	84 DAT
Untreated	nd	nd
Abamectin/Trunk Injection	nd	nd
Abamectin/Airblast	nd	nd
